# Ranked determinants of telemedicine diabetic retinopathy screening performance in the United States primary care safety-net setting: an exploratory CART analysis

**DOI:** 10.1186/s12913-022-07915-5

**Published:** 2022-04-14

**Authors:** S. Lee Ware, Christina R. Studts, Feitong Lei, Heather Bush, Eric B. Higgins, Jamie L. Studts, Ana Bastos de Carvalho

**Affiliations:** 1 Department of Ophthalmology and Visual Sciences, University of Kentucky College of Medicine, 110 Conn Terrace, Ste 550, Lexington, KY 40508 USA; 2 Department of Pediatrics, University of Colorado School of Medicine, Aurora, CO USA; 3 Department of Biostatistics, University of Kentucky College of Public Health, Lexington, KY USA; 4 Cancer Prevention and Control Program, University of Colorado Cancer Center, Aurora, USA; 5 Division of Medical Oncology, Department of Medicine, University of Colorado Anschutz Medical Campus, Aurora, CO USA

**Keywords:** Diabetic retinopathy, Primary care, Telemedicine, Screening, Barriers and facilitators, Determinants, Classification and regression tree (CART), Consolidated framework for implementation research (CFIR), Mixed methods, Underserved

## Abstract

**Background:**

Diabetic retinopathy (DR) is a leading cause of blindness worldwide, despite easy detection and effective treatment. Annual screening rates in the USA remain low, especially for the disadvantaged, which telemedicine-based DR screening (TDRS) during routine primary care has been shown to improve. Screening rates from such programs have varied, however, pointing to inconsistent implementation and unaddressed barriers. This work seeks to identify and prioritize modifiable barriers for targeted intervention.

**Methods:**

In this final phase of an exploratory mixed-methods study, we developed, validated, and administered a 62-item survey to multilevel stakeholders involved with TDRS in primary care safety-net clinics. Survey items were aligned with previously identified determinants of clinic-level screening and mapped to the Consolidated Framework for Implementation Research (CFIR). Classification and Regression Tree (CART) analyses were used to identify and rank independent variables predictive of individual-level TDRS screening performance.

**Results:**

Overall, 133 of the 341 invited professionals responded (39%), representing 20 safety-net clinics across 6 clinical systems. Respondents were predominately non-Hispanic White (77%), female (94%), and between 31 and 65 years of age (79%). Satisfaction with TDRS was high despite low self-reported screening rates. The most important screening determinants were: provider reinforcement of TDRS importance; explicit instructions by providers to staff; effective reminders; standing orders; high relative priority among routine diabetic measures; established TDRS workflows; performance feedback; effective TDRS champions; and leadership support.

**Conclusions:**

In this survey of stakeholders involved with TDRS in safety-net clinics, screening was low despite high satisfaction with the intervention. The best predictors of screening performance mapped to the CFIR constructs *Leadership Engagement*, *Compatibility*, *Goals & Feedback*, *Relative Priority*, *Champions*, and *Available Resources*. These findings facilitate the prioritization of implementation strategies targeting determinants of TDRS performance, potentially increasing its public health impact.

**Supplementary Information:**

The online version contains supplementary material available at 10.1186/s12913-022-07915-5.

## Background

Diabetic retinopathy (DR) remains the leading cause of blindness among working-age adults in the USA [1], despite its easy detection and the widespread availability of effective treatment. The American Diabetes Association recommends annual DR screening for all diabetics — a service traditionally delivered through in-person specialist exam — but screening rates remain low [2], especially among disadvantaged populations disproportionately served by safety-net clinics such as Federally Qualified Health Centers (FQHC) [3, 4]. While single-purpose specialist visits for screening are rife with known barriers to access, most persons with diagnosed diabetes visit their primary care provider at least once per year [5]. Telemedicine-based DR screening (TDRS) embedded in the primary care setting and delivered as an important part of routine diabetes care — a modality proven to increase DR screening rates [6, 7] — can remove the known barriers to compliance [8–11] and increase early detection of vision-threatening pathologies [12], all while providing cost savings, especially for low-income populations and rural patients with high transportation costs [13].

While the extent of TDRS adoption among FQHCs in the US is unknown, the implementation, screening performance, and sustainability of primary care-based TDRS programs so far published have been mixed [14–19]. Yet few studies have investigated the determinants of program screening rates, fewer have correlated perceived barriers with measures of effectiveness, and fewer still have rigorously investigated how to systematically improve TDRS implementation.

Implementation strategies are “methods or techniques used to enhance the adoption, implementation, and sustainability of a clinical program or practice,” like TDRS [20]. The knowledge base for implementation strategies is growing and suggests that multilevel, multicomponent implementation strategies that target context-specific barriers and facilitators [21, 22] to intervention adoption, delivery, and sustainment may have the greatest impact on implementation success [23, 24].

Our previous work described a large FQHC-based TDRS network’s creation, policies, screening performance, and sustainment [6], and, using key informant interviews, reported the barriers and facilitators of program implementation perceived by multi-level professionals engaged in TDRS delivery, emphasizing those that distinguish higher- from lower-screening programs [25]. However, both our work and the literature so far lack a quantitative assessment of the relative importance of specific TDRS determinants in the safety-net setting.

Through this final phase of our sequential exploratory mixed-methods research approach, we sought (1) to quantify personnel and program characteristics, perceptions of TDRS delivery, and expectations of potential implementation strategies among multilevel stakeholders in the primary care safety-net setting; (2) to reconcile these findings with the implementation determinants identified in earlier phases of our research; (3) to organize our findings within an actionable theoretical framework; and (4) to prioritize them as a foundation for future implementation mapping [26]. This work is therefore a valuable contribution to our understandings of the interplay among real-world conditions, intervention characteristics, and implementation strategies for TDRS delivery in the primary care safety-net setting.

## Methods

The Strengthening the Reporting of Observational Studies in Epidemiology (STROBE) checklist guided this report (Additional file 1). Because this manuscript reports only the quantitative results of a sequential exploratory study, we believe the manuscript to be better served by a reporting guideline for observational studies, such as STROBE, rather than one intended for mixed methods reports. For example, though not explicitly discussed in the manuscript, we did consider Creamer’s criteria of interpretive comprehensiveness as critical to our Synthesis of Multiphase Findings in the Discussion section. Likewise, we found “the quality of mixed methods studies in health services research” assessments for quantitative components, integration, and insights to be helpful.

We developed a novel survey instrument to quantify perceptions regarding program barriers and facilitators at multiple professional strata within our TDRS network (for more information on the network’s implementation and characteristics, please see our earlier work [6]). Based on results from key informant interviews obtained during our study’s qualitative phase [25] and a survey of relevant literature, items tapping specific characteristics of the intervention, practice setting, and population of interest were generated to measure stakeholder perceptions of TDRS program implementation, as well as expectations regarding the potential effects of proposed implementation strategies.

To assess face and content validity prior to instrument distribution, cognitive interviews were performed with an ophthalmologist, an ophthalmic nurse, a telemedicine specialist, two implementation researchers, and three lay reviewers whose feedback helped refine response options; the relevance and quality of each item; and the overall clarity, organization, and scope of the instrument. Conventional pretesting, that is, rehearsal piloting in the manner and mode intended for the final survey administration, was also used to identify technical defects, frequency distributions, average time to completion, and other aspects of the survey’s administration and reporting.

The final survey instrument included 62 items addressing contextual factors serving as potential barriers and facilitators to the implementation of TDRS, personal experience with and opinions regarding TDRS, and finally, demographic and clinical practice setting characteristics using a combination of multiple choice, Likert scales, and multimodal scale response options (Additional file 2). The target time to completion for respondents was 10 min.

The importance of coherence among determinants, interventions, and theory is well established [27]. In their extensive review of models used to study mHealth adoption, Jacob et al. [28] noted that the Consolidated Framework for Implementation Research (CFIR) — a contextual framework of theoretical domains and constructs associated with effective implementation of clinical innovations [29] — was a more comprehensive tool than the more widely used Technology Acceptance Model (TAM), the diffusion of innovation theory (DOI), and the unified theory of acceptance and use of technology (UTAUT) models, particularly in the areas of monetary factors, user experience, organizational factors, workflow characteristics, and policy and regulation factors. Citing an emerging consensus, they go on to endorse the use of frameworks that, like the CFIR, can accommodate the impact of barriers to implementation at the levels of individual behavior, the complexity of health care institutions and practices, and the policy and regulatory environments in which healthcare is delivered.

To increase construct validity and to orient future selection of implementation strategies, each survey item was mapped to one or more relevant constructs of the meta-theoretical CFIR. Two implementation researchers (ABC and SLW) independently mapped instrument items to CFIR constructs based on the descriptions and rationale provided by the CFIR Research Team at the Center for Clinical Management Research. Mappings were reconciled through an iterative process of discussion and expert consultation (CRS), resulting in a final consensus crosswalk between survey items and the CFIR.

For example, the CFIR Inner Setting construct *Goals & Feedback* — which reflects “The degree to which goals are clearly communicated, acted upon, and fed back to staff, and alignment of that feedback with goals” — was related to two questionnaire items, both of which posed hypotheticals of whether respondents believed they would be more likely to order or perform TDRS if they were given more data as feedback. A full description of the CFIR crosswalk is included in the Supplemental Methods section of Additional file 3.

After receiving ethical approval from the university’s Institutional Review Board (IRB#44107) and participatory commitment from the leaderships of network clinics, we built the validated survey instrument using Qualtrics^XM^ (Qualtrics, Provo, UT). The sampling frame for this survey included all of our network’s clinical employees directly involved with their clinic’s TDRS program. Participation was voluntary, and completion was incentivized (10.00 USD). Disclosure prefaced the instrument, followed by screening items to identify respondents’ profession and clinical role, and to confirm TDRS involvement. All respondents were presented with a core set of 41 items, and the 21 remaining items varied based on the respondent’s reported clinical role: provider or staff.

In October 2019, survey links were distributed by clinic- or system-level administrators to all providers and staff involved in their TDRS program. Eligible staff were primarily medical assistants performing tasks such as eligibility checks, charting, and the performance of the TDRS exam itself. Up to three reminders were sent by clinic directors over the four-week collection window, which closed in November 2019.

### Statistical analysis

Analyses were first conducted to describe the distribution of responses. Bivariate associations with professional strata were investigated using chi-square tests of independence.

Using *screening performance* (i.e., “Of all your telemedicine diabetic eye screening-eligible patients during the past 12 months, what percentage did you screen?”) as the categorical outcome of interest, we looked for bivariate associations with independent variables by dividing respondents into two groups: *lower screening* (those selecting “0-25%” from the response options) and *higher screening* (those selecting “25-50%”, “51-75%”, or “more than 75%”). “Unsure” responses (18) were treated as missing and excluded from the analysis. This lower versus higher dichotomy was chosen based on the network’s low overall screening rates, which were well below the national average and recognized targets [2]. Logistic regression was performed to identify associations between independent variables (e.g., professional strata, the presence of an established TDRS workflow, etc.) and membership in the *higher screening* group, and reported as estimated odds ratios (with 95% confidence intervals). Statistical significance was determined as *p* < .05 (two-tailed) for all tests. 

Targeting implementation strategies in limited-resource settings requires the identification of determinants involved and their prioritization by degree of influence on program performance [30]. To explore and visualize the variable interactions associated with screening performance, we performed exploratory Classification and Regression Tree (CART) analyses. CART analysis is an atheoretical non-parametric exploratory technique. Through a process of recursive partitioning, CART analysis can account for higher-order interactions among independent variables, accommodates small sample sizes [31], multicollinearity [32], and incomplete datasets [33], and produces both classification trees and variable importance rankings useful for prioritizing targets of intervention without implying causal relations [34]. The generated classification tree consists of parent and binary child nodes iteratively and recursively split upon the independent variable that best reduces the variability in the dependent variable. Each subsequent split beyond the root node reflects higher-order interactions. CART analysis also produces a variable importance ranking (VIR) that reflects the relative importance of each independent variable to the construction of the final tree (calculated as the change in model-predicted values per change in the independent variable’s value), regardless of whether the variable is used to split a parent node. The VIR is therefore a powerful tool for measuring and comparing the overall influence of predictor variables on the outcome of interest, and provides a more complete picture than the decision tree alone can convey [35] by accounting for variable masking [36]. In the development of implementation strategies, such a ranking of determinants by strength of association with intervention performance may be of greater value than the final decision tree itself [37, 38].

To better visualize the multifaceted relationships among variables representing modifiable determinants with the potential to influence TDRS performance (Table S1), CART analysis was employed as an exploratory method [39]. Although CARTs are typically used to probe large data sets, the application here provides a preliminary strategy to (1) visualize the variable interactions associated with screening performance, and to (2) assess the relative importance of each variable to screening performance. The CART analyses were restricted to those variables considered to represent modifiable determinants, i.e., those amenable to change by targeted implementation strategies. The primary CART analysis was limited to only those items delivered to both providers and staff. Secondary CART analysis forced the first breakpoint by professional stratum, and included variables unique to each professional role (Table S1). We utilized the Gini impurity function to determine optimal splits, and, because this exploratory method sought to “rule in” variables, trees were pruned according to the maximum difference in risk, defined as 0 standard errors. Respondents with missing values (*n* = 8) were included in the CART using surrogate variables [40]. CART analyses were performed using SPSS (IBM SPSS Statistics for Windows, Version 27.0. Armonk, NY: IBM Corp).

## Results

The survey link was sent to 341 employees of 20 clinics representing 6 safety-net clinical systems, of whom 133 (39%) submitted responses — 36 providers and 97 staff. Respondents were predominately non-Hispanic White (77%), female (94%), and between 31 and 65 years of age (79%). Staff were distinguished from providers by differences in gender (*p* = .002) and ethnicity (*p* = .038); by length of time involved with TDRS (*p* = .030); and by practical knowledge of their clinic’s TDRS program (operationalized by identifying the type of camera used, *p* = .004; Table 1).

**Table 1:** Respondent demographic, professional, and intervention-specific characteristics by professional stratum

Respondent, Clinic Characteristics (n)	Providers (%)	Staff (%)	***P***-value*
**Gender**			.002
Female (119)	29 (83)	90 (98)	
Male (8)	6 (17)	2 (2)	
**Age**			.739
18-30 years (22)	4 (11)	18 (20)	
31-45 years (51)	14 (40)	37 (40)	
46-65 years (49)	16 (46)	33 (36)	
> 65 years (4)	1 (3)	3 (3)	
Prefer not to specify (1)	0	1 (1)	
**Race / Ethnicity**			.038
White, non-Hispanic (98)	32 (91)	66 (71)	
Black or African American (11)	0	11 (12)	
Hispanic or Latino (10)	0	10 (11)	
Asian (6)	3 (9)	3 (3)	
American Indian or Alaska native (1)	0	1 (1)	
Prefer not to specify (1)	0	1 (1)	
**Education**			N/A
High school diploma (1)	0	1 (1)	
Some college (21)	0	21 (23)	
2-year degree (50)	0	50 (54)	
4-year degree (7)	0	7 (8)	
Professional degree (32)	22 (63)	10 (11)	
Doctorate (13)	13 (37)	0	
Prefer not to specify (3)	0	3 (3)	
**Professional experience**			.968
0-5 years (46)	12 (34)	34 (37)	
6-10 years (26)	7 (20)	19 (21)	
11-20 years (19)	6 (17)	13 (14)	
More than 20 years (36)	10 (29)	26 (28)	
**Time with organization**			.645
< 1 year (23)	6 (17)	17 (18)	
1-5 years (55)	17 (47)	38 (39)	
6-10 years (23)	7 (19)	16 (16)	
> 10 years (32)	6 (17)	26 (27)	
**Length of experience with TDRS**			.030
< 1 year (60)	9 (25)	51 (54)	
1-2 years (25)	9 (25)	16 (17)	
3-5 years (43)	16 (44)	27 (28)	
> 5 years (3)	2 (6)	1 (1)	
**TDRS camera type used**			.004
Desktop/Tabletop (80)	23 (64)	57 (59)	
Handheld/Portable (33)	3 (8)	30 (31)	
Both (7)	2 (6)	5 (5)	
Unsure (13)	8 (22)	5 (5)	

*Abbreviations*: *TDRS* telemedicine diabetic retinopathy screening

*P-values were generated from chi-square test

When asked to estimate their personal screening performance over the preceding 12 months, more than one third of respondents (39%) reported screening ≤25% of their eligible patients, and the majority (57%) screened fewer than half of those eligible. While the majority of respondents ordered/performed TDRS at least once per week (59%), nearly a quarter ordered/performed less than one screening per month (23%). Paradoxically, 95% of respondents reported overall satisfaction with TDRS (Table 2).

**Table 2:** Responses to key measures of TDRS utilization by professional stratum

Survey Item, responses (n)	Providers (%)	Staff (%)
What percentage of eligible patients did you screen?		
0-25% (51)	11 (31)	40 (42)
26-50% (24)	4 (11)	20 (21)
51-75% (19)	7 (19)	12 (13)
> 75% (20)	10 (28)	10 (10)
Unsure (18)	4 (11)	14 (15)
How frequently do you use TS for your patients?		
Daily (29)	7 (19)	22 (23)
Weekly (48)	13 (36)	35 (37)
Monthly (23)	9 (25)	14 (15)
Less than once per month (30)	7 (19)	23 (24)
Please rate your overall satisfaction with TS.		
Very satisfied (41)	13 (36)	28 (30)
Satisfied (83)	22 (61)	61 (65)
Dissatisfied (6)	1 (3)	5 (5)
What percentage of your diabetic patients do you think got their recommended screening for diabetic eye disease in the last year [whether in your clinic through TS, or elsewhere by an eye care provider]?		
0-25% (33)	6 (17)	27 (28)
26-50% (33)	5 (14)	28 (29)
51-75% (44)	21 (58)	23 (24)
> 75% (11)	4 (11)	7 (7)
Unsure (11)	0	11 (11)

*Abbreviations*: *TDRS and TS* telemedicine diabetic retinopathy screening

Further, when comparing respondents who reported a screening rate (i.e., did not select “unsure” from the item response options), providers and staff differed significantly (*p* = .008). The majority of providers (53%) reported screening > 50% of eligible patients, while only 27% of staff reported doing so. Similarly, when considering the broader question of whether their patients were being screened at all (through in-clinic TDRS, or by outside eyecare specialists), providers and staff again had significantly different perceptions (*p* = .003). Most providers (69%) believed that the majority (> 50%) of their patients were being screened, compared to 35% of staff believing so.

Figure S1 shows survey response distributions and survey item alignment with the CFIR domains Process, Intervention Characteristics, and Characteristics of Individual*s*. Figures. S2 and S3 show the survey response distributions and survey item alignments for constructs in the Inner Setting domain (see Additional file 4).

Staff were generally comfortable performing the intervention, favorable of its characteristics and time required, satisfied with the provided training, and reported high levels of leadership direction to perform the intervention.

Providers were more likely to be white and male and to have worked with TDRS longer, but were less likely to be familiar with the TDRS-specific equipment. Providers also perceived gradeability less favorably than other intervention characteristics.

Both professional strata perceived intervention champions as effective catalysts for TDRS, though they were reportedly present in only a minority of clinics. Reminders were appreciated when present, and, along with established workflows and increased staffing, were considered very likely to improve future screening performance if implemented.

Associations with *screening performance*, the categorical outcome of interest, were assessed of all survey items common to both providers and staff, as well as those specific to providers or staff. The following items were associated with greater odds of screening: more than 2 years of experience with TDRS, at least monthly use; patient objection as the primary reason not to screen; standing orders; explicit positive instructions (for staff only); staff autonomy to perform the intervention (staff only); and effective reminders, workflows, and champions. Running behind (for providers only) and perception of low patient adherence to screening recommendations were associated with lower odds of screening (Table 3).

**Table 3:** Variables significantly associated with screening performance

Variable Label (n)	LS Group *n* = 51 (%)	HS Group *n* = 63 (%)	OR	95% CI	***P***-value^**#**^
Experience with TDRS?					0.008
> 2 years (40)	11 (27.5)	29 (72.5)	3.1	1.4—7.1	
2 or fewer years (74)	40 (54.0)	34 (46.0)	REF		
Frequency of use?					<.0001
At least weekly (72)	20 (27.8)	52 (72.2)	16.5	4.4—61.8	
Monthly (19)	11 (57.9)	8 (42.1)	4.6	1.0—21.1	
Less than once per month (22)	19 (86.4)	3 (13.6)	REF		
Why not: Patient Objection?					0.0003
No (50)	32 (64.0)	18 (36.0)	0.2	0.1—0.5	
Yes (64)	19 (29.7)	45 (70.3)	REF		
Champion: present, effective?					0.0545
Yes, very effective (29)	8 (27.6)	21 (72.4)	2.0	0.8—5.3	
Yes, moderately effective or ineffective (16)	11 (68.8)	5 (31.2)	0.3	0.1—1.0	
Not present (64)	28 (43.8)	36 (56.2)	REF		
Are patients compliant with screening guidelines?					<.0001
0-25% (29)	23 (79.3)	6 (20.7)	0.1	0.0—0.2	
26-50% (29)	13 (44.8)	16 (55.2)	0.3	0.1—0.9	
> 50% (50)	11 (22.0)	39 (78.0)	REF		
Effective reminder/alert in place?					0.0101
Yes (59)	19 (32.2)	40 (67.8)	2.8	1.3—6.0	
No (51)	29 (56.9)	22 (43.1)	REF		
Standing order?					0.0024
Unsure (21)	17 (81.0)	4 (19.0)	0.2	0.1—0.7	
Yes (63)	21 (33.3)	42 (66.7)	1.5	0.6—3.8	
No (26)	11 (42.3)	15 (57.7)	REF	REF	
Workflow present?					0.0014
Yes (77)	26 (33.8)	51 (66.2)	3.9	1.7—9.1	
No (36)	24 (66.7)	12 (33.3)	REF		
^a^Explicit positive instructions?					0.0554
Always/Very frequently (58)	23 (39.7)	35 (60.3)	6.8	1.4—34.6	
Rarely/Never (22)	15 (68.2)	7 (31.8)	REF		
^a^Staff autonomy?					0.0485
Yes (51)	20 (39.2)	31 (60.8)	2.6	1.0—6.9	
No (27)	17 (63.0)	10 (37.0)	REF		
^b^Why not: Running behind?					0.0053
No (11)	3 (27.3)	8 (72.7)	7.9	1.2—51.8	
Yes (21)	13 (61.9)	8 (38.1)	REF		

*Abbreviations*: *TDRS* telemedicine diabetic retinopathy screening, *LS* lower screening, *HS* higher screening, *OR* estimated odds ratio, *CI* confidence interval

^a^Responses restricted to staff only (*n* = 82)

^b^Responses restricted to providers only (*n* = 32)

^#^*P*-values were obtained from univariate logistic regression model

**Fig. 1:**
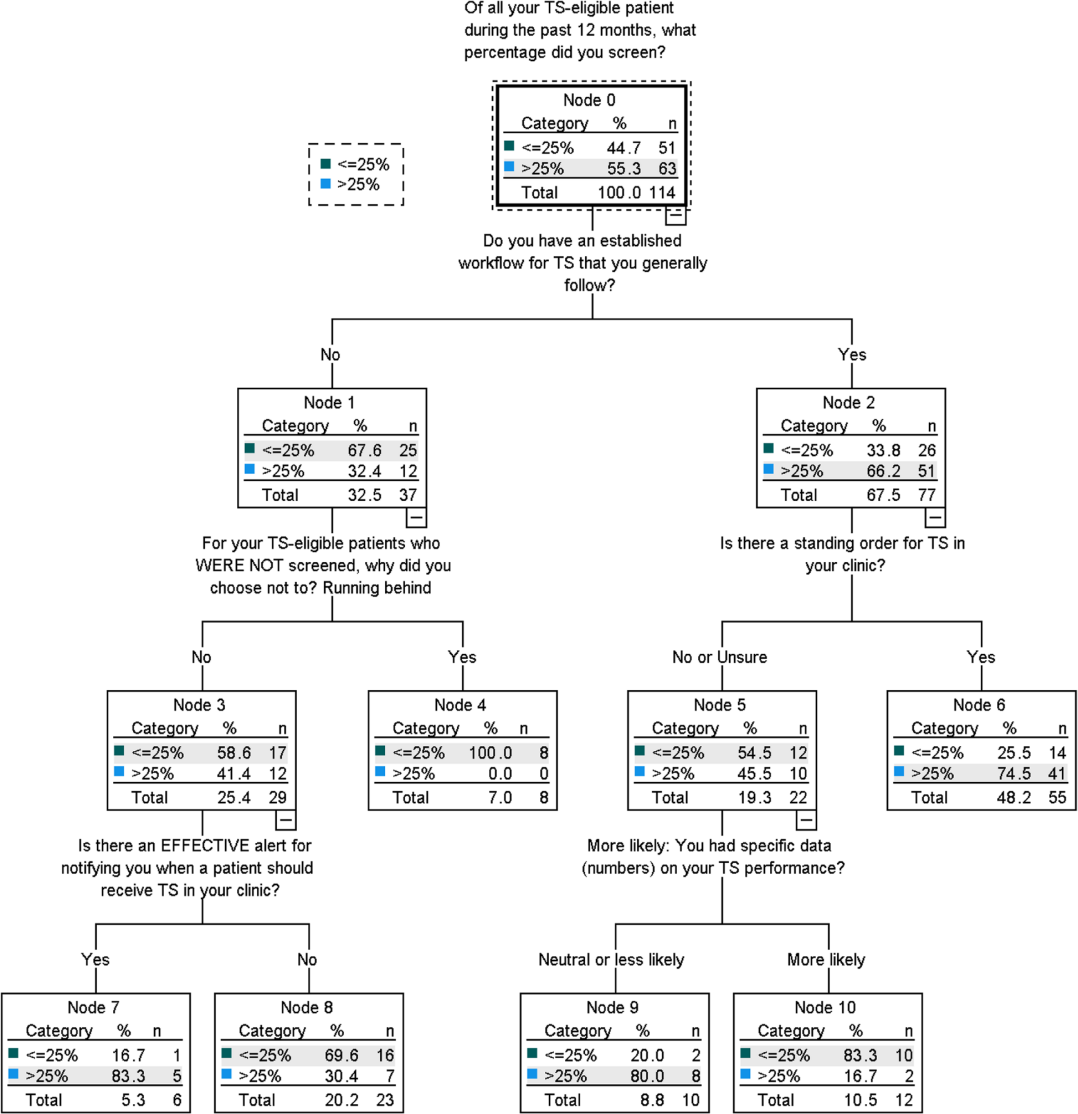
Primary classification tree for modifiable determinants of TDRS performance. Abbreviations: TDRS and TS, telemedicine diabetic retinopathy screening

The primary CART analysis produced a VIR for independent predictors of individual-level TDRS performance. Eight variables were considered important to the model (importance value ≥.01): effective alerts (.058); standing orders (.057); established workflows (.047); access to performance data (.040); effective champions (.039); access to comparative performance data (.030); encouragement from leadership (.020); and failure to screen due to running behind (.019). The corresponding classification tree (Fig. 1) identified five predictors whose interactions best distinguished lower from higher screeners: (1) established workflow, (2) running behind, (3) standing orders, (4) effective alerts, and (5) expected effect of performance feedback. “Established workflow” best split the full sample (114 cases) — those with an established TDRS workflow being more likely to screen. Those without an established workflow were best split by “running behind” as the reason for not screening, and then by the presence of effective alerts. For those with an established workflow, the presence of standing orders best distinguished higher screeners. In the absence of standing orders for TDRS, lower screeners were more likely to value the proposition of increased performance data. The pruned primary CART model’s accuracy for predicting a respondent’s screening performance was 77.2% (*r* = .228, SE = .039), 66.7% for those screening ≤25% of eligible patients, and 85.7% for those designated higher screeners (> 25% of eligible patients). Based on its risk estimate, we consider the model a good fit for the data.

Secondary CART analysis, which included profession-specific variables and forced the first tree break by professional stratum, identified variables involving interprofessional communication as the most important predictors for both providers (.151) and staff (.076). Other variables important to the secondary model included providers’ perceptions of TDRS priority (.051), the effectiveness of champions (.034), the presence of alerts (.024), and providers’ explicit instructions not to perform TDRS (.011). The secondary model’s overall accuracy was lower than the primary model’s (69.3%; *r* = .307, SE = .043), better predicting higher screeners than lower (85.7% vs 49.0%, respectively).

## Discussion

In this final phase of a sequential exploratory mixed-methods study, we developed, validated, and delivered a 62-item survey to multilevel stakeholders involved with TDRS delivery in primary care safety-net clinics. Survey items were aligned with implementation determinants and were mapped to the CFIR for construct validity, to enable cross-study comparisons, and to inform future implementation mapping. Logistic regression and exploratory CART analyses were used to identify the variables most strongly associated with individual-level screening performance.

While most TDRS studies have focused on patient satisfaction, we found that overall satisfaction of professionals involved with TDRS was high, despite low performance rates. Though acceptability is the most commonly assessed implementation outcome [41], the discrepancy noted here suggests that post-implementation acceptability of the intervention is insufficient to drive and sustain consistent use, i.e., penetration and sustainability [42]. This is consistent with our hypothesis of multiple interacting implementation determinants, and reinforces the importance of comprehensive multi-level program assessment [43].

Patient objection was the most cited (50%) reason for not screening eligible patients. Patients may object to TDRS for many reasons (lack of time, lack of trust, competing health problems, lack of symptoms, recent but undocumented screening, etc. [8, 9, 25, 44]), and because this variable was not further refined within the instrument, it was not included in CART analyses. From its correlation with higher screening performance, we interpreted the selection of “patient objection” to indicate the relative absence of other barriers. Other reasons cited, such as “short staffed” and “running behind”, confirmed our earlier findings and agreed with the conclusions of Ogunyemi et al., who cite staff shortages, disruptions, and diversions [45], and the qualitative results described by Liu et al., who cite time and resource constraints [46]. “Running behind” was also a critical predictor of lower screening performance in this study among those without an established workflow.

### Synthesis of multiphase findings

Evidence for direct correlations between perceived barriers and intervention performance is critical to implementation planning [47] and strategy selection [26], yet lacking in the literature for TDRS. Addressing this gap, the previous qualitative phase of this study found associations between clinic-level TDRS performance and the six CFIR constructs *Available Resources*, *Relative Priority*, *Leadership Engagement*, *Goals & Feedback*, *Engaging*, and *Champions* [25].

**Fig. 2:**
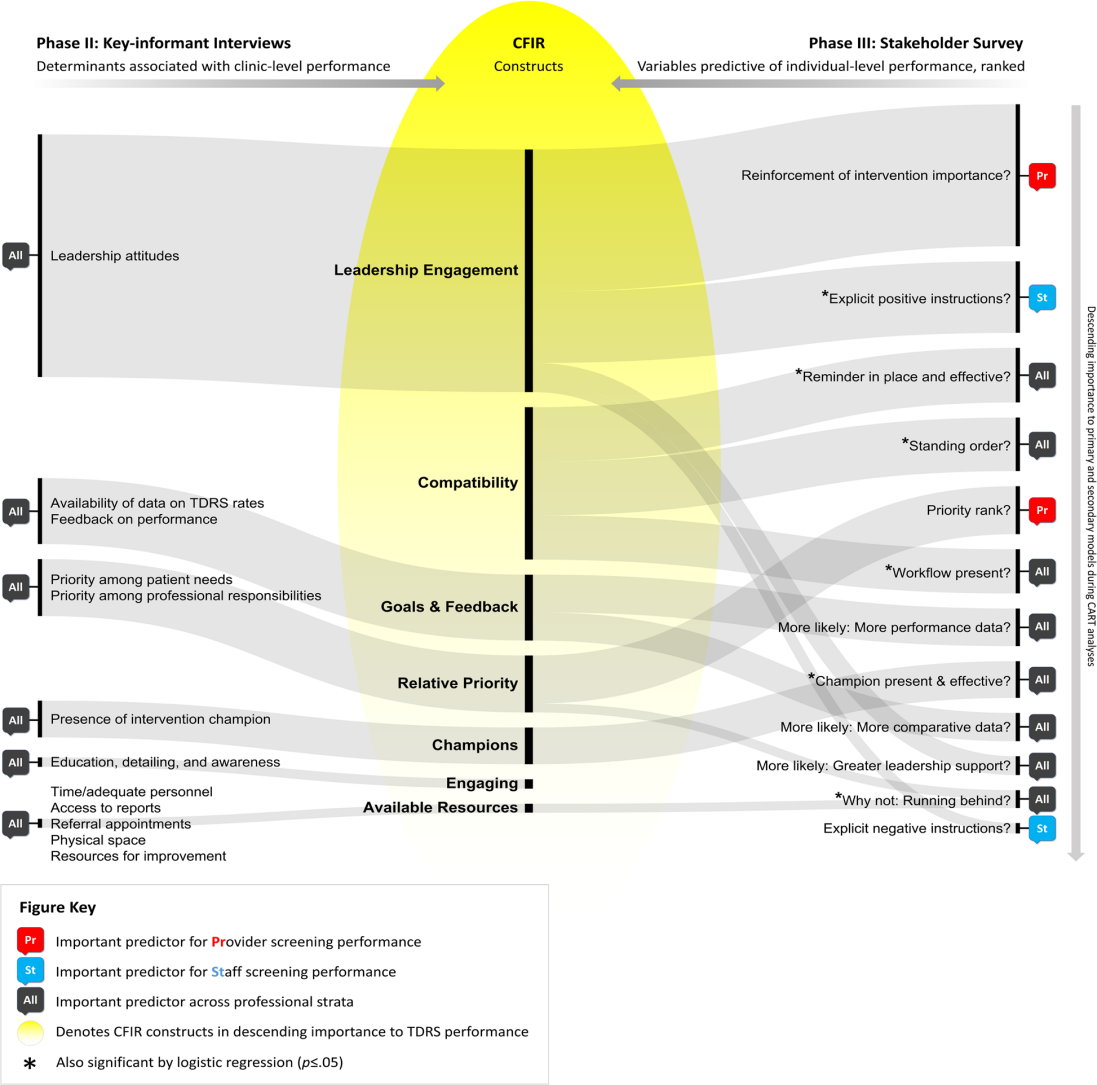
Convergence of determinants associated with TDRS performance upon aligned CFIR constructs. Abbreviations: CFIR, Consolidated Framework for Implementation Research; TDRS, telemedicine diabetic retinopathy screening; CART, Classification and Regression Tree; VIR, variable importance ranking. The Phase III variable ranking was determined from each variable’s VIR value in the primary or secondary CART model

Building on those findings, our current work corroborates, expands, and preliminarily prioritizes the list of candidate barriers and facilitators of TDRS performance in the safety-net setting. By using exploratory CART analyses to rank-order modifiable determinants, we have taken a significant step towards the development and prioritization of targeted implementation strategies [48] aimed to maximize impact in a safety-net setting defined by its resource constraints. This is a novel approach for dissemination and implementation research, which we intend to further explore and develop through future studies. Figure 2 illustrates the synthesis of our qualitative and quantitative findings.

In CART analyses, variables with high importance values are the drivers of the outcome measure. Since screening performance is the outcome of complex, interacting, multi-level factors, we should not expect to find a single variable with a predominant importance value in our dataset. VIR provides a ranking of ratio values based on the contribution of each predictor to the model. In our results, the importance values for several variables were very similar, indicating that these variables equally contributed to the model and that no single variable was the obvious driver of the categorical outcome measure.

The construct *Leadership Engagement*, which was strongly associated with clinic-level TDRS performance in our qualitative interview data [25], was endorsed by some of the most important predictors of individual-level screening performance in the current study. In secondary CART analysis, provider-initiated interprofessional communication best predicted screening performance among providers *and* among staff, suggesting that, given their roles as clinical decision-makers and influencers, provider buy-in and reinforcement is a first-order priority in TDRS implementation and in improvement and sustainment plans. In a quality improvement study, Liu and colleagues used the NIATx framework to engage clinical leaders in participatory and iterative TDRS program improvement, which resulted in a sustained increase in DR screening rates [49]. Similarly, Ogunyemi et al. noted that, “support from high-level administration and leadership [ …] was instrumental in the most successful clinic implementations,” adding that such leadership engagement increased the likelihood of both initiating and troubleshooting TDRS among personnel involved [45].

Significantly, three of the six most important determinants of individual-level screening performance in our CART models (which were also significant in logistic regression) mapped to the CFIR construct *Compatibility* (effective reminders, standing orders, and established workflows). A fourth determinant that mapped to *Compatibility*, staff autonomy (i.e., “Are you allowed to perform TDRS in eligible patients without a verbal request from the provider or an order put in by the provider?”) — while not prominent in CART regressions — was significant in logistic regression. This cluster of determinants is an important addition to our understanding of TDRS program implementation and performance, as *Compatibility* was not emphasized by our prior qualitative data. The AAO underlines the importance of workflow adaptation and workflow metrics by which to monitor and improve TDRS integration [50], points echoed by Bouskill and colleagues in describing the “squeeze approach” for TDRS when implemented without adequate workflow redesign [51]. They identified several critical vulnerabilities within TDRS workflows, the first being breakdowns in the processes of identifying, recruiting, and handing off patients for screening, findings congruent with our *Compatibility*-mapped predictors. Liu et al., based on qualitative stakeholder interviews, noted similar barriers relating to *Compatibility*, and proposed strategies to streamline TDRS workflow processes, such as the adoption of effective electronic health record reminders [46]. To our knowledge, standing orders and staff autonomy have not elsewhere been elucidated as important factors in TDRS success, yet may be critical buoys of screening performance in the absence of consistent, explicit positive orders by providers.

Aspects of the construct *Goals & Feedback* were found to be predictors of clinic- and individual-level TDRS performance, especially relating to performance feedback and access to comparative TDRS performance data. This is an insight not apparent from the raw survey results (since neither of these survey items garnered more than 50% endorsement of expected positive effect by respondents) nor from the tests of independence, thus highlighting the value of CART techniques to identify higher-order interactions among independent variables (e.g., lower screeners with established workflows but without standing orders were more likely to respond that the provision of performance feedback would improve their screening performance).

Tracing back to the importance of the communication behavior of opinion leaders in Rogers’ DOI [52], the importance of intervention champions to successful evidence-based intervention (EBI) implementation has been well established [53]. Champions have been proven critical change agents for the primary care setting [54], and at least one study has demonstrated champions’ importance to telehealth services implementation and sustainment [55]. Our work is the first to establish evidence of champion effectiveness for teleophthalmology screening. Champions were qualitatively associated with intervention promotion, timely resource mobilization, and increased communication among professionals in our key informant interview data [25], and their importance to individual-level screening performance was further demonstrated here.

The CFIR constructs *Relative Priority* and *Available Resources* were significant in both our prior qualitative and current quantitative datasets. For providers, variables representing *Relative Priority* and *Leadership Engagement* were interactive (i.e., among providers who inconsistently or infrequently communicated the importance of TDRS to staff, those who valued DR screening equal to HbA1c measures were more likely to be higher screeners than those who valued DR screening less than HbA1c measures).

While our prior key informant interviews identified several determinants aligned with the construct *Available Resources* and associated with clinic-level screening performance — many of which were subsequently included in the survey instrument — only one was predictive of individual-level screening performance in our survey: “running behind”. Similarly, Mamillapalli et al. found that the most commonly cited limitation of TDRS performance In a private practice setting was “availability of staff [ …] and extra time consumed to perform the eye exams” [56]. In fact, limited resources have featured prominently in most reports on barriers to TDRS. Running behind, which mapped to both *Available Resources* and *Relative Priority*, reflects the natural consequence of what Bouskill et al. [51] described as “new burdens on already-strapped safety-net clinics.” From their qualitative study of staff workarounds for TDRS in the safety-net setting, they concluded that “the additional needs identified by new screening processes, when not met through additional follow-up resources, leave frontline staff in the uncomfortable position of having to witness inequality and resource constraints without the ability to systematically address them.” This is a critical conclusion that connects downstream integration and performance barriers to upstream failures during the implementation process [48], and highlights the precarious circumstances into which EBIs like TDRS must be implemented and to which they must be adapted through careful pre-implementation planning and resource allocation if their potential patient benefits are to be realized.

Though associated with clinic-level TDRS performance in our earlier qualitative data (e.g., professional education, detailing, and awareness), the CFIR construct *Engaging* was not endorsed by our survey as significant to individual-level screening performance. It is possible that *Engaging* was underrepresented in the survey.

### Limitations

Because the response rate was limited and we lacked access to demographic information on those who did not participate, we were unable to assess the potential impacts of selection and participation biases. Also, because the study was cross-sectional and exploratory, we were unable to determine causal relationships. Our reliance on self-reported screening performance as the dependent variable is a limitation, though the dimensional expansion from a clinic-level to individual-level screening performance measure, coupled with the parsimonious convergence with our prior qualitative findings, buffers our confidence. Additionally, residual confounding due to unmeasured variables is a potential limitation, though our sequential mixed-methods design, which allowed exploratory qualitative data to inform the survey’s composition, and instrument mapping to the CFIR mitigated this risk as much as was possible. Our dataset lacked sufficient power for model validation by confirmatory machine learning, and while the CART models were stable in sensitivity analysis, findings here must be considered exploratory. Despite these limitations, we have identified, contextualized, and preliminarily ranked by importance the modifiable determinants of TDRS performance in the primary care safety-net setting, which, upon confirmatory testing, can inform the development of targeted, evidence-based implementation strategies to increase screening rates.

## Conclusions

In this survey of multi-level stakeholders involved with TDRS in safety-net clinics, post-implementation acceptability, measured as satisfaction with the intervention, was high even while overall screening performance lagged. Several variables were found to be associated with higher TDRS performance, which substantiated and expanded our prior insights. Together, our triangulated multiphase mixed methods results emphasize barriers and facilitators aligned with the CFIR constructs *Leadership Engagement*, *Compatibility*, *Goals & Feedback*, *Champions*, *Engaging*, *Relative Priority*, and *Available Resources* as the key determinants of TDRS program screening performance.

## Supplementary Information

**Additional file 1.** Identifies locations of key information included in the manuscript.

**Additional file 2.** Includes the complete survey instrument utilized in this study.

**Additional file 3. Supplementary Methods.** Describes mapping of survey items to the Consolidated Framework for Implementation Research domains and constructs.

**Additional file 4. Supplementary Figures and Tables.** Includes supplementary figures for survey response distributions organized by domain and construct.

## Data Availability

The dataset analyzed during the current study is available from the corresponding author by reasonable request. This is an open access article distributed in accordance with the Creative Commons Attribution Non Commercial (CC BY-NC 4.0) license, which permits others to distribute, remix, adapt, build upon this work non-commercially, and license their derivative works on different terms, provided the original work is properly cited, appropriate credit is given, any changes made indicated, and the use is non-commercial. See: http://creativecommons.org/licenses/by-nc/4.0/.

## Abbreviations

DR: Diabetic retinopathy
FQHC: Federally qualified health centers
TDRS: Telemedicine diabetic retinopathy screening
CFIR: Consolidated Framework for Implementation Research
CART: Classification and Regression Tree
VIR: Variable importance ranking
EBI: Evidence-based intervention
STROBE: Strengthening the Reporting of Observational Studies in Epidemiology
TAM: Technology Acceptance Model
DOI: Diffusion of innovation theory
UTAUT: Unified theory of acceptance and use of technology
IRB: Institutional review board
