# Supplementary material for: Ranked determinants of telemedicine diabetic retinopathy screening performance in the United States primary care safety-net setting: an exploratory CART analysis
Source: BMC Health Serv Res. 2022 Apr 14;22:507. doi: 10.1186/s12913-022-07915-5 (PMC9011929; doi:10.1186/s12913-022-07915-5)
Supplement: Supplementary file 2 — Additional file 2. Includes the complete survey instrument utilized in this study. [file 12913_2022_7915_MOESM2_ESM.docx]

TDRS IS: Cross Validation

Start of Block: Informed assent

Researchers at the University of Kentucky are inviting you to take part in this survey about screening of diabetic eye disease because you have a role in this screening at your work.

Although you may not get personal benefit from taking part in this research study, your responses may help us understand more about diabetic retinopathy screening via telemedicine.  Some volunteers experience satisfaction from knowing they have contributed to research that may possibly benefit others in the future.

Your response to the survey is anonymous, which means no names will appear or be used on research documents or be used in presentations or publications.  The research team will not know that any information you provided came from you, nor even whether you participated in the study.

You will be paid $10.00 USD for taking part in this study.

The survey will take about 8 minutes to complete.

There are no known risks from participating in this study.

You have a choice about whether or not to complete the survey. If you do participate, you are free to skip any questions or discontinue at any time.

Please be aware that, while we make every effort to safeguard your data once received on our servers via Qualtrics (the survey software company), given the nature of online surveys, we can never guarantee the confidentiality of the data while still en route to us.

If you have questions about the study, please feel free to ask. My contact information is given below. If you have complaints, suggestions, or questions about your rights as a research volunteer, contact the staff in the University of Kentucky Office of Research Integrity at (859) 257-9428 or toll-free at 1-866-400-9428.

To ensure your responses will be included, please complete and submit your survey within 2 weeks of receiving the invitation email.

Thank you in advance for your assistance with this important project to help patients who are living with diabetes.

Sincerely,

Ana Carvalho

Dept. of Ophthalmology/College of Medicine, University of Kentucky

PHONE:  859-323-5267

E-MAIL: aba253@uky.edu

If you would like to participate, please select the "I agree to participate" radio button below.

- I agree to participate

End of Block: Informed assent

Start of Block: Clinical Role

The clinic where you work is equipped with a device used to perform **screening of diabetic eye disease via telemedicine**. In this survey, each time we refer to **diabetic eye disease Telemedicine Screening**, we will use the term "TS".

Please select your clinical role regarding TS:

- Provider (MD, DO, NP, PA)
- Staff Member

End of Block: Clinical Role

Start of Block: Current Practice

How long have you been working in this clinic (or group of clinics)?

- Less than 1 year
- 1-5 years
- 6-10 years
- Over 10 years

How many patients do you see in a typical week?

- 1-25
- 26-50
- 51-75
- More than 75 patients

On average, what percentage of the patients you see in a typical week are diabetic?

- 0-25%
- 26-50%
- 51-75%
- More than 75%
- Unsure

On a priority list, where does diabetic eye disease screening fall for you relative to all routine diabetic exams?

- Most important
- Equally important to HbA1c
- Less important than HbA1c, but equal to foot exam and kidney function testing
- Less important than HbA1c, foot exam or kidney function testing

What percentage of your diabetic patients do you think got their recommended screening for diabetic eye disease in the last year (whether in your clinic through TS, or elsewhere by an eye care provider)?

- 0-25%
- 26-50%
- 51-75%
- More than 75%
- Unsure

What type of TS imaging device is currently used in your clinic?

- Desktop/Tabletop
- Handheld/Portable
- Both (desktop and handheld)
- Unsure what type is used

To the best of your knowledge, how long has your clinic been using TS?

- Less than 1 year
- 1-2 years
- 3-5 years
- More than 5 years

How long have you personally been using TS for your patients?

- Less than 1 year
- 1-2 years
- 3-5 years
- More than 5 years

How frequently do you use TS for your patients?

- Daily
- Weekly
- Monthly
- Less than once per month

For the next two questions, consider a TS-eligible patient to be any diabetic patient presenting in clinic who has NOT received diabetic eye disease screening (via TS or with an eye care provider) in 12 months or more.

Of all your TS-eligible patients during the past 12 months, what percentage did you screen?

- 0-25%
- 26-50%
- 51-75%
- More than 75%
- Unsure

For your TS-eligible patients who WERE NOT screened, why did you choose not to? (please select all that apply)

- Short staffed
- Running behind
- Patient objection
- Believe TS to be inferior to in-person screening by eye care specialist
- It's a low priority exam
- Other - please specify ________________________________________________

Please rate your overall satisfaction with TS.

- Very satisfied
- Satisfied
- Dissatisfied
- Very dissatisfied

Please rate TS on the following attributes:

|  | Good | Acceptable | Poor |
| --- | --- | --- | --- |
| Camera software |  |  |  |
| Image acquisition |  |  |  |
| Quality of training received |  |  |  |
| Time it takes to perform |  |  |  |
| Camera location in clinic |  |  |  |
| Fit in clinic workflow |  |  |  |
| Convenience (for you) |  |  |  |
| Rate of ungradable images |  |  |  |
| Quality of the report |  |  |  |

When was the last time you received training for TS?

- Within the last year
- Between 1 and 2 years ago
- More than 2 years ago

Please rate the training  you received for TS in the following aspects:

|  | Good | Acceptable | Poor | Did not receive this training |
| --- | --- | --- | --- | --- |
| How to use the software |  |  |  |  |
| How to use the camera |  |  |  |  |
| How to acquire images |  |  |  |  |
| Distinguishing between good and bad quality images |  |  |  |  |
| Troubleshooting equipment |  |  |  |  |
| Troubleshooting an eye where you're getting a bad quality image |  |  |  |  |

End of Block: Current Practice

Start of Block: Communication

For the following questions, please respond based on the provider you work with most.

Does the provider instruct you to perform TS on eligible patients?

- Always
- Very frequently
- Half the time
- Rarely
- Never

Does the provider instruct you NOT to perform TS on eligible patients?

- Always
- Very frequently
- Half the time
- Rarely
- Never

Separate from ordering, how often do you communicate to your staff the importance of performing TS?

- With each eligible patient
- Daily
- Weekly
- Monthly
- Annually
- Never

End of Block: Communication

Start of Block: Facilitators

Do you have an established workflow for TS that you generally follow?

- Yes
- No

How do you think having a set workflow would influence your use of TS?

- Increase use of TS
- No effect on use of TS
- Decrease use of TS

Is there a standing order in your clinic for TS?

- Yes
- No
- Unsure

Are you allowed to perform TS in eligible patients without a verbal request from the provider or an order put in by the provider?

- Yes
- No

Is there an EFFECTIVE alert for notifying you when a patient should receive TS in your clinic?

- Yes
- No

How much more likely would you be to use TS if there were an EFFECTIVE alert method?

- More likely
- No effect
- Less likely

What would be an EFFECTIVE TS alert method for you? (please select all that apply)

- EMR flag placed by scrubber
- Automated EMR alert when exam is due
- Automated order when exam is due
- Other - Please describe what you think would work best: ________________________________________________

Is there a champion for TS in your clinic (someone who strongly advocates for TS)?

- Yes
- No

In your opinion, how effective has the champion been in increasing use of TS in your clinic?

- Very effective
- Moderately effective
- Not effective

How much more likely would you be to use TS if:

|  | More likely | No effect | Less likely |
| --- | --- | --- | --- |
| ⊗The provider put in a TS order for the patient (instead of using a standing order by default)? |  |  |  |
| You received more training with TS? |  |  |  |
| More clinic staff were available to work with you? |  |  |  |
| The TS exams were performed on the visit day but outside of your clinic by a TS-trained professional? (e.g., a diabetes educator, radiology tech, pharmacy or lab tech, etc.) |  |  |  |
| The TS reports were integrated into the EMR? |  |  |  |
| You had more encouragement from your leadership regarding TS? |  |  |  |
| You had specific data (numbers) on your TS performance? |  |  |  |
| You had data comparing performance between you and your colleagues? (data wouldn't reveal identities of colleagues) |  |  |  |
| For patients with a positive screening, referrals to an eye care specialist were handled by an external patient coordinator? (e.g., from the University of Kentucky) |  |  |  |

End of Block: Facilitators

Start of Block: Demographics

You're almost done. To finish, please share some basic demographic information with us.

Gender

- Male
- Female
- Other
- Prefer not to specify

Age

- 18-30 years old
- 31-45 years old
- 46-65 years old
- Over 65 years old
- Prefer not to specify

Race / Ethnicity (please select all that apply)

- White
- Black or African American
- American Indian or Alaska Native
- Asian
- Native Hawaiian or Pacific Islander
- Hispanic or Latino
- Other ________________________________________________
- Prefer not to specify

Highest education level completed

- Less than high school
- High school diploma or equivalent
- Some college
- 2-year degree
- 4-year degree
- Professional degree
- Doctorate
- Prefer not to specify

How long have you been in your current profession (regardless of employer)?

- 0-5 years
- 6-10 years
- 11-20 years
- More than 20 years

What is your practice setting? (Select all that apply)

- Small private group practice (5 or fewer practitioners)
- Large private group practice (6 or more practitioners)
- Academic practice
- Hospital-based clinic
- Managed care organization
- Federally-qualified health center (FQHC)
- Other ________________________________________________

End of Block: Demographics

Start of Block: Block 7

Thank you for completing the survey. Please click the "next" arrow below to submit your survey and be redirected to provide us your contact information  so we can compensate your time with $10.00 USD. 


Your contact information will NOT be linked to your survey responses. Your contact information will be kept confidential and will only be used to send you the $10.00 USD.

End of Block: Block 7
