# Supplementary material for: Ranked determinants of telemedicine diabetic retinopathy screening performance in the United States primary care safety-net setting: an exploratory CART analysis
Source: BMC Health Serv Res. 2022 Apr 14;22:507. doi: 10.1186/s12913-022-07915-5 (PMC9011929; doi:10.1186/s12913-022-07915-5)
Supplement: Supplementary file 3 — Additional file 3. Supplementary Methods. Describes mapping of survey items to the Consolidated Framework for Implementation Research domains and constructs. [file 12913_2022_7915_MOESM3_ESM.pdf]

Supplemental File 3

Supplemental Methods

Survey-to-CFIR crosswalk

In summary, five items aligned with the CFIR domain Characteristics of Individuals and addressed barriers related to respondents' perceptions of patient preference, as well as respondents' comfort performing the screening procedure. Five items aligned with the CFIR's Intervention Characteristics domain, addressing determinants such as access to screening reports, the time efficiency of the screening process, and the ease with which respondents could acquire images of sufficient quality. Three items aligned with the domain Process. These addressed determinants such as education and detailing intended to increase awareness of the evidence base for the intervention, as well as the presence and effect of intervention champions.

The majority of survey items aligned with the CFIR domain Inner Setting. Eight items were mapped to the Inner Setting's *Access to Knowledge & Information* construct and addressed issues of intervention training and support. Two items related to performance data and aligned with the CFIR's *Goals & Feedback* construct. The CFIR's *Leadership Engagement* construct and the determinant "leadership attitudes" were addressed by four of the survey's items.

The Inner Setting construct *Compatibility* was broadly addressed. It aligned with thirteen survey items across five of the twenty-one determinants identified during the study's qualitative phase ("physical space for equipment", "reminder to perform", "method of clinical order request", "workflow", and "difficulty obtaining referral appointments"). Seven items were mapped through three determinants to the construct *Available Resources*, and three aligned with the *Relative Priority* construct and two distinct priority-related determinants.

No items aligned with the Outer Setting CFIR domain.

Some items aligned with secondary and tertiary constructs, while several items — such as those addressing demographics and post-implementation performance — did not align with any construct in the CFIR.
