# Supplementary material for: Ranked determinants of telemedicine diabetic retinopathy screening performance in the United States primary care safety-net setting: an exploratory CART analysis
Source: BMC Health Serv Res. 2022 Apr 14;22:507. doi: 10.1186/s12913-022-07915-5 (PMC9011929; doi:10.1186/s12913-022-07915-5)
Supplement: Supplementary file 4 — Additional file 4. Supplementary Figures and Tables. Includes supplementary figures for survey response distributions organized by domain and construct. [file 12913_2022_7915_MOESM4_ESM.pdf]

Supplementary figures and tables

Table S1. Survey items as independent variables representing modifiable determinants for CART analyses, with mapped CFIR constructs.

**Item Label (independent variable)**	**Modifiable Determinant(s)**	**CFIR Construct(s)**
Why not: Short Staffed?	Time / adequate personnel	Available Resources
Why not: Running behind?	Time / adequate personnel; Priority among patient needs	Available Resources; Relative Priority
Why not: Low regard for TDRS?	Leadership attitudes; Education, detailing, and awareness	Access to Knowledge & Information; Engaging
Why not: Low priority exam?	Time / adequate personnel; Priority among responsibilities	Available Resources; Relative Priority
Workflow present?	Workflow	Compatibility
Standing order?	Method of clinical order request; Workflow	Compatibility
Reminder in place and effective?	Reminder to perform	Compatibility
Champion present and effective?	Presence of intervention champion	Champions
More likely: Integrated reports?	Access to screening reports; Workflow;	Available Resources; Compatibility; Design Quality
More likely: Greater leadership support?	Leadership attitudes	Leadership Engagement
More likely: More performance data?	Availability of performance data	Goals & Feedback
More likely: More comparative data?	Availability of performance data	Goals & Feedback
More likely: Patient coordinator?	Difficulty obtaining referral appointments	Available Resources; Compatibility
*Priority rank?	Priority among patient needs	Relative Priority
*Leadership reinforcement?	Leadership attitudes	Leadership Engagement
*More likely: on-site auxiliary service?	Time / adequate personnel; Workflow	Available Resources; Compatibility
**Explicit positive instructions?	Leadership attitudes	Leadership Engagement
**Explicit negative instructions?	Leadership attitudes	Leadership Engagement
**Staff autonomy?	Method of clinical order request; Workflow	Compatibility
**More likely: Provider order?	Method of clinical order request; Workflow	Compatibility
**More likely: More training?	Training and support; Comfort performing screenings	Access to Knowledge & Information; Self-Efficacy
**More likely: More staff?	Time / adequate personnel;	Available Resources

Legend
Abbreviations: CART, Classification and Regression Tree; CFIR, Consolidated Framework for Implementation Research; TDRS, telemedicine diabetic retinopathy screening.
*Provider-only item; **Staff-only item.

Figure S1. Results mapped to the CFIR domains Characteristics of Individuals, Intervention Characteristics, and Process.

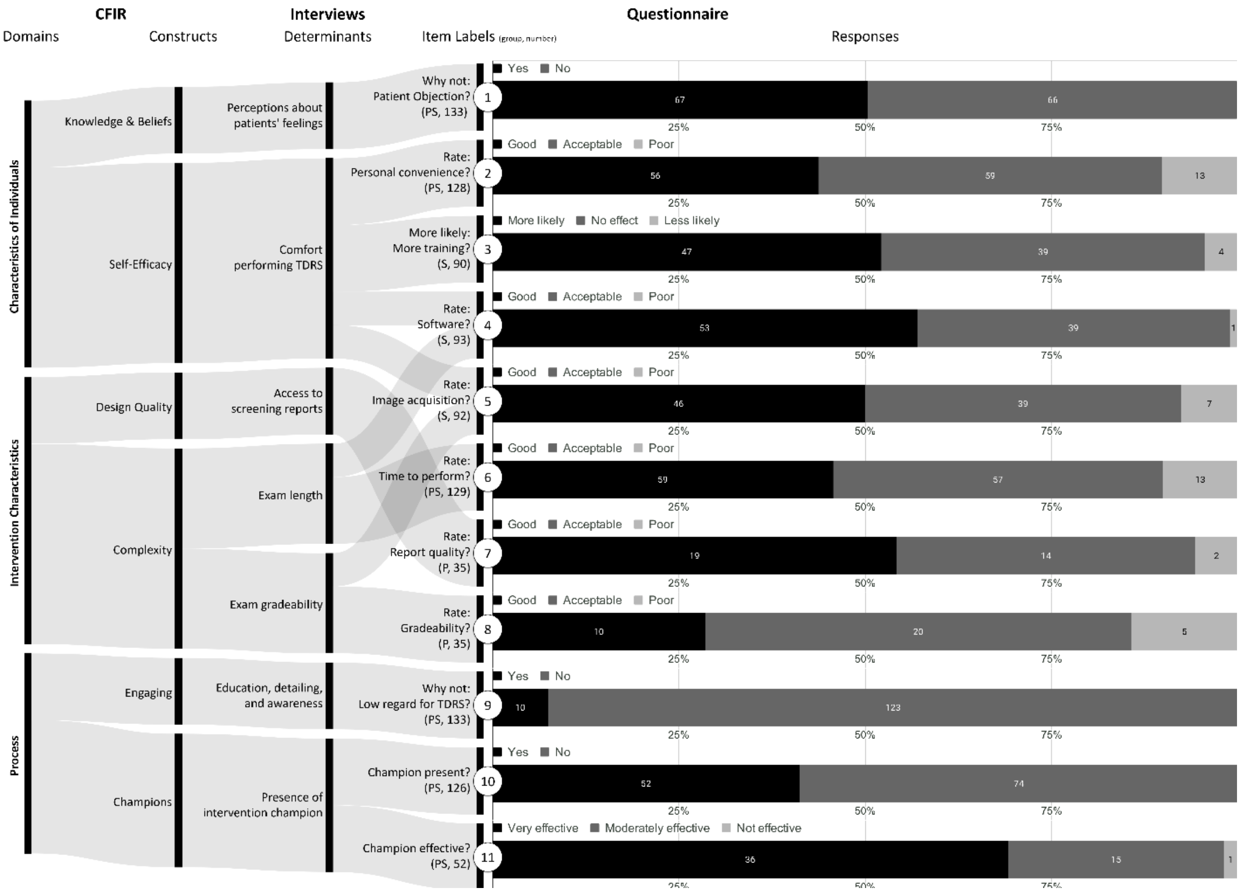

Legend

CFIR, Consolidated Framework for Implementation Research; TDRS and TS, telemedicine diabetic retinopathy screening; P, Providers; S, Staff.

1. For your TS-eligible patients who WERE NOT screened, why did you choose not to? (please select all that apply) — Patient objection
2. Please rate TS on the following attributes: — Convenience (for you)
3. How much more likely would you be to use TS if: — You received more training with TS?
4. Please rate TS on the following attributes: — Camera software
5. Please rate TS on the following attributes: — Image acquisition
6. Please rate TS on the following attributes: — Time to perform
7. Please rate TS on the following attributes: — Report quality
8. Please rate TS on the following attributes: — Gradeability
9. For your TS-eligible patients who WERE NOT screened, why did you choose not to? (please select all that apply) — Believe TS to be inferior to in-person screening by eye care specialist
10. Is there a champion for TS in your clinic (someone who strongly advocates for TS)?
11. In your opinion, how effective has the champion been in increasing use of TS in your clinic?

Figure S2. Results mapped to the CFIR Inner Setting constructs *Access to Knowledge & information*, *Goals & Feedback*, and *Leadership Engagement*.

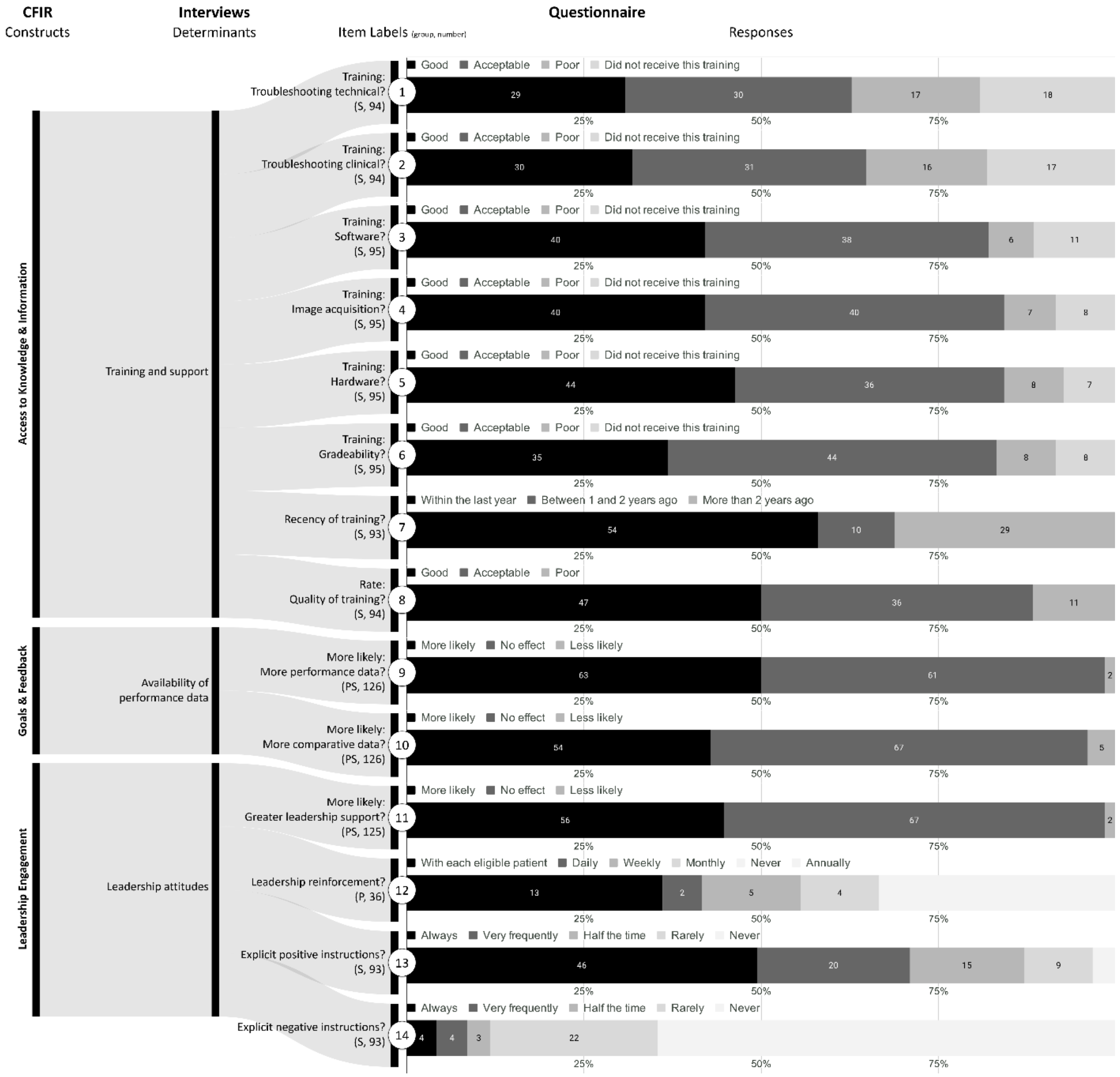

Legend

CFIR, Consolidated Framework for Implementation Research; TDRS and TS, telemedicine diabetic retinopathy screening; P, Providers; S, Staff.

1. Please rate the training you received for TS in the following aspects: - Troubleshooting equipment
2. Please rate the training you received for TS in the following aspects: - Troubleshooting an eye where you're getting a bad quality image
3. Please rate the training you received for TS in the following aspects: - How to use the software
4. Please rate the training you received for TS in the following aspects: - How to acquire images
5. Please rate the training you received for TS in the following aspects: - How to use the camera
6. Please rate the training you received for TS in the following aspects: - Distinguishing between good and bad quality images
7. When was the last time you received training for TS?
8. Please rate TS on the following attributes: - Quality of training received
9. How much more likely would you be to use TS if: - You had specific data (numbers) on your TS performance?
10. How much more likely would you be to use TS if: - You had data comparing performance between you and your colleagues? (data

wouldn't reveal identities of colleagues)

11. How much more likely would you be to use TS if: - You had more encouragement from your leadership regarding TS?
12. Separate from ordering, how often do you communicate to your staff the importance of performing TS?
13. Does the provider instruct you to perform TS on eligible patients?
14. Does the provider instruct you NOT to perform TS on eligible patients?

Figure S3. Results aligned with the CFIR Inner Setting constructs *Compatibility*, *Available Resources*, and *Relative Priority*.

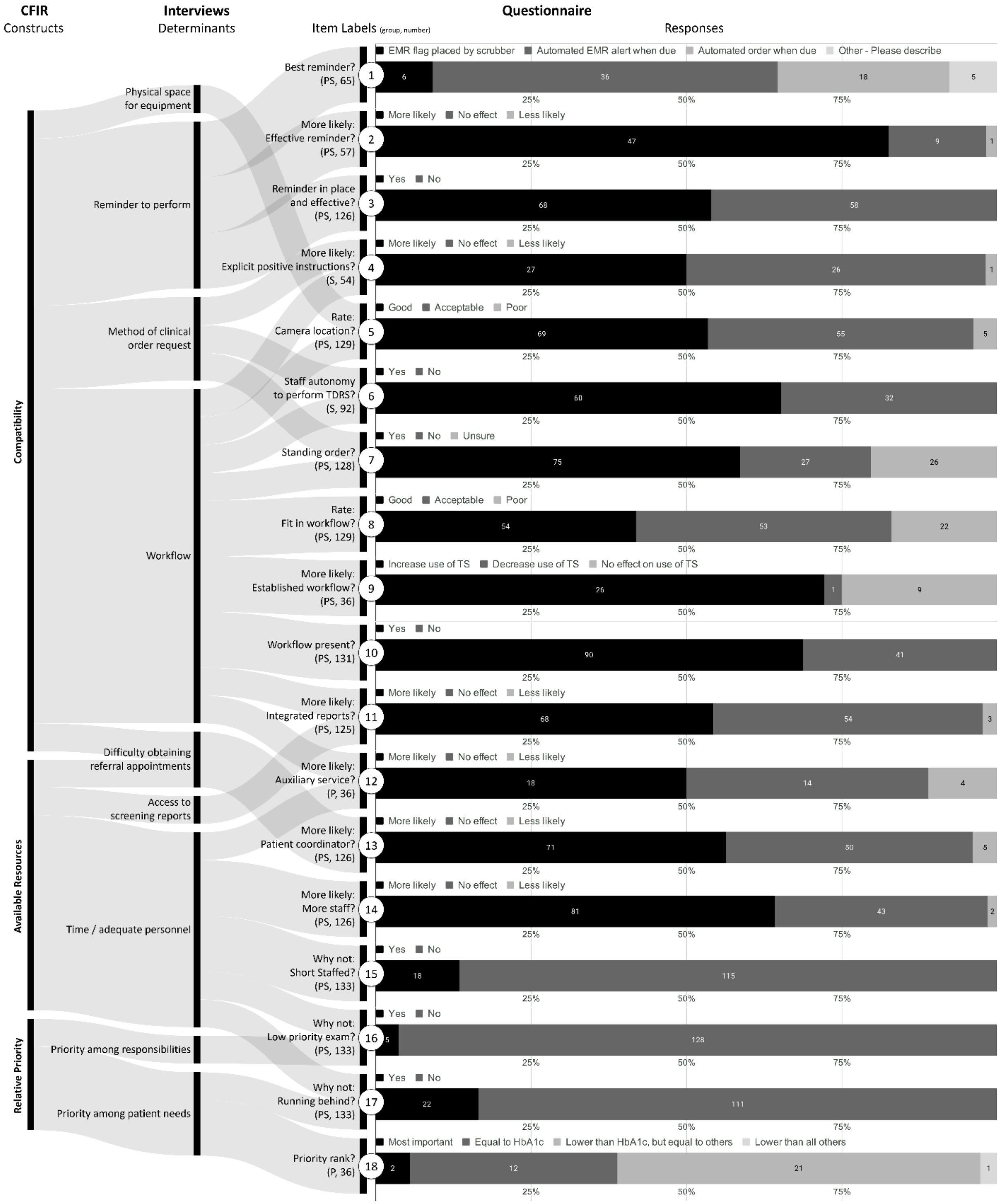

Legend

CFIR, Consolidated Framework for Implementation Research; TDRS and TS, telemedicine diabetic retinopathy screening; P, Providers; S, Staff.

1. What would be an EFFECTIVE TS alert method for you? (please select all that apply)
2. How much more likely would you be to use TS if there were an EFFECTIVE alert method?

3. Is there an EFFECTIVE alert for notifying you when a patient should receive TS in your clinic?
4. How much more likely would you be to use TS if: - The provider put in a TS order for the patient (instead of using a standing order by default)?
5. Please rate TS on the following attributes: - Camera location in clinic
6. Are you allowed to perform TS in eligible patients without a verbal request from the provider or an order put in by the provider?
7. Is there a standing order for TS in your clinic?
8. Please rate TS on the following attributes: - Fit in clinic workflow
9. How do you think having a set workflow would influence your use of TS?
10. Do you have an established workflow for TS that you generally follow?
11. How much more likely would you be to use TS if: - The TS reports were integrated into the EMR?
12. How much more likely would you be to use TS if: - The TS exams were performed on the visit day but outside of your clinic by a TS-trained professional? (e.g., a diabetes educator, radiology tech, pharmacy or lab tech, etc.)
13. How much more likely would you be to use TS if: - For patients with a positive screening, referrals to an eye care specialist were handled by an external patient coordinator? (e.g., from the University of Kentucky)
14. How much more likely would you be to use TS if: - More clinic staff were available to work with you?
15. For your TS-eligible patients who WERE NOT screened, why did you choose not to? (please select all that apply) - Short staffed
16. For your TS-eligible patients who WERE NOT screened, why did you choose not to? (please select all that apply) - It's a low priority exam
17. For your TS-eligible patients who WERE NOT screened, why did you choose not to? (please select all that apply) - Running behind
18. On a priority list, where does diabetic eye disease screening fall for you relative to all routine diabetic exams?
